# Supplementary material for: Overwintering of Thrips (Thysanoptera) Under the Bark of the Plane Tree (Platanus x hispanica Mill. ex Münchh.) in Southeastern Poland
Source: Insects. 2025 Jan 17;16(1):92. doi: 10.3390/insects16010092 (PMC11765657; doi:10.3390/insects16010092)
Supplement: Supplementary file 1 [file insects-16-00092-s001.zip › insects-3390572-supplementary.pdf]

Supplementary Materials

Table S1. Data on the sites, species, and dates of thrips collection.

Lp.	Site	No. of Specimens	No. of Species	GPS Coordinates	Date of Collection
1.	Bachórzec	2	2	49.83° (N) 22.33° (E)	09.03.2014
2.	Bolestraszyce	7	6	49.82° (N) 22.86° (E)	21.03.2014
3.	Czudec	19	3	49.95° (N) 21.84° (E)	13.04.2014
4.	Dubiecko	20	6	49.83° (N) 22.39° (E)	06.03.2014
5.	Dzikowiec	111	6	50.32° (N)21.89° (E)	06.04.2015
6.	Jasionka	4	2	50.11° (N)22.06° (E)	23.02.2014
7.	Klemensów	32	3	50.43° (N) 23.01° (E)	30.03.2014
8.	Kombornia	7	2	49.70° (N) 21.87° (E)	22.03.2014
9.	Krasiczyn	2	1	49.78° (N) 22.65° (E)	09.03.2014
10.	Łopuszka Mała	90	1	49.97° (N) 22.41° (E)	13.03.2014
11.	Medyka	73	2	49.81° (N) 22.93° (E)	13.04.2014
12.	Miejsce Piastowe	19	1	49.63° (N) 21.79° (E)	11.03.2014
13.	Nisko	8	3	50.52° (N) 22.14° (E)	23.03.2014
14.	Pełkinie	2	1	50.05° (N) 22.65° (E)	07.03.2015
15.	Przemyśl	3	2	49.81° (N) 22.76° (E)	02.04.2015
16.	Przeworsk	5	4	50.06° (N) 22.48° (E)	09.03.2014
17.	Roźwienica	5	4	49.95° (N) 22.60° (E)	12.04.2014
18.	Rzeszów	12	4	50.05° (N) 22.01° (E)	09.03.2014
19.	Słocina	16	2	50.03° (N) 22.04° (E)	02.03.2014
20.	Tarnów	6	1	50.00° (N) 21.01° (E)	13.03.2014
21.	Urzejowice	2	2	50.01° (N)22.46° (E)	14.12.2014
22.	Werynia	2	1	50.24° (N)21.81° (E)	10.03.2014
23.	Wzdów	9	2	49.64° (N) 22.00° (E)	23.03.2014
24.	Wola Sękowa	3	3	49.51° (N)22.00° (E)	21.03.2014
25.	Zaczernie	2	2	50.09° (N) 22.00° (E)	11.03.2014
26.	Zasów	3	2	50.13° (N) 21.32° (E)	24.02.2016
27.	Zawada	21	5	49.98° (N) 21.01° (E)	20.03.2014
28	Żarnowiec	4	4	49.69° (N)21.67° (E)	08.03.2014
29.	Żuklin	3	3	49.93° (N) 22.43° (E)	14.03.2014
